# A case of long-term survival treated with three metastasectomies and two subsequent adjuvant nivolumab therapies for recurrent malignant melanoma of the esophagus

**DOI:** 10.1186/s40792-022-01561-z

**Published:** 2022-11-04

**Authors:** Keita Hanada, Shigeru Tsunoda, Motoo Nomura, Shintaro Fujimura, Yojiro Yutaka, Tatsuto Nishigori, Shigeo Hisamori, Hisatsugu Maekewa, Nobuaki Hoshino, Atsushi Itami, Eiji Tanaka, Kazutaka Obama

**Affiliations:** 1 Department of Surgery, Graduate School of Medicine, Kyoto University, 54 Shogoin- Kawahara-Cho, Sakyo-Ku, Kyoto, 606-8507 Japan; 2 Department of Clinical Oncology, Kyoto University Hospital, 54 Shogoin- Kawahara-Cho, Sakyo-Ku, Kyoto, 606-8507 Japan; 3 Department of Otolaryngology-Head and Neck Surgery, Graduate School of Medicine, Kyoto University, 54 Shogoin- Kawahara-Cho, Sakyo-Ku, Kyoto, 606-8507 Japan; 4 Department of Thoracic Surgery, Graduate School of Medicine, Kyoto University, 54 Shogoin- Kawahara-Cho, Sakyo-Ku, Kyoto, 606-8507 Japan; 5 Department of Surgery, Kobe City Nishi-Kobe Medical Center, 5-7-1 Kojidai, Nishi-Ku, Kobe, 651-2273 Japan; 6 Department of Gastroenterological Surgery and Oncology, Kitano Hospital Medical Research Institute, Ohgimachi, Kita-Ku, Osaka, 530-8480 Japan

**Keywords:** Primary malignant melanoma of the esophagus, Metastasis, Recurrence, Nivolumab

## Abstract

**Background:**

The treatment strategy for metastatic lesions of primary malignant melanoma of the esophagus (PMME) is currently determined on a case-by-case basis, based on the National Comprehensive Cancer Network (NCCN) guidelines for cutaneous melanoma. The NCCN guidelines state that resection should be considered in patients with resectable metastatic recurrence. Herein, we report a case of long-term survival treated with three metastasectomies and two subsequent adjuvant nivolumab therapies for the metastatic recurrence of PMME.

**Case presentation:**

A 65-year-old female patient with PMME underwent thoracoscopic subtotal esophagectomy, gastric tube reconstruction via the posterior mediastinal route, and cervical esophagogastric anastomosis. Histopathological examination of the resected specimen revealed that the tumor was PMME with tumor invasion into the muscularis propria and no lymph node metastasis. At the age of 68 years, she developed intestinal invagination due to jejunal metastasis of malignant melanoma and underwent resection of the jejunum. Histopathological examination of the resected specimen revealed two metastases of malignant melanoma in the jejunum and one metastasis to the mesenteric lymph node. At the age of 75 years, a recurrence of malignant melanoma was found in the cervical esophagus. She underwent thoracoscopic mobilization of the gastric tube and esophagus followed by cervical esophagectomy and reconstruction with a free jejunum flap. She received 24 courses of nivolumab therapy for 1 year as a postoperative adjuvant therapy. Subsequently, at the age of 78 years, an enlarged left cervical lymph node and a mass in the right lower lobe of the lung were found. She underwent left cervical lymph node dissection and thoracoscopic wedge resection of the right lung. Histopathological examination of the resected specimens revealed that both tumors were metastases of malignant melanoma. At age 79 years, she received eight courses of nivolumab therapy as a second postoperative adjuvant therapy, with no sign of recurrence in a 9-month follow-up period after the third metastasectomy.

**Conclusion:**

In cases of metastatic recurrence of PMME, aggressive resection of oligometastasis with postoperative adjuvant nivolumab therapy may result in long-term survival.

## Background

Primary malignant melanoma of the esophagus (PMME) is a rare disease with poor prognosis [1–3]. Recently, the efficacy of immune checkpoint inhibitors (such as anti-programmed death 1 (PD-1) antibodies) for cutaneous melanoma has been reported in clinical trials [4], and the National Comprehensive Cancer Network (NCCN) guidelines for cutaneous melanoma recommend their use in metastatic and unresectable cases [5]. The use of anti-PD-1 antibodies for PMME has also been reported [1, 6–8]. The treatment strategy for PMME is currently determined on a case-by-case basis, based on the NCCN guidelines for cutaneous melanoma [5], the United Kingdom (UK) national guidelines for head and neck mucosal melanoma [9], and other relevant guidelines. Herein, we report a case of long-term survival treated with three metastasectomies and two subsequent adjuvant nivolumab therapies for the metastatic recurrence of PMME.

## Case presentation

**Fig. 1:**
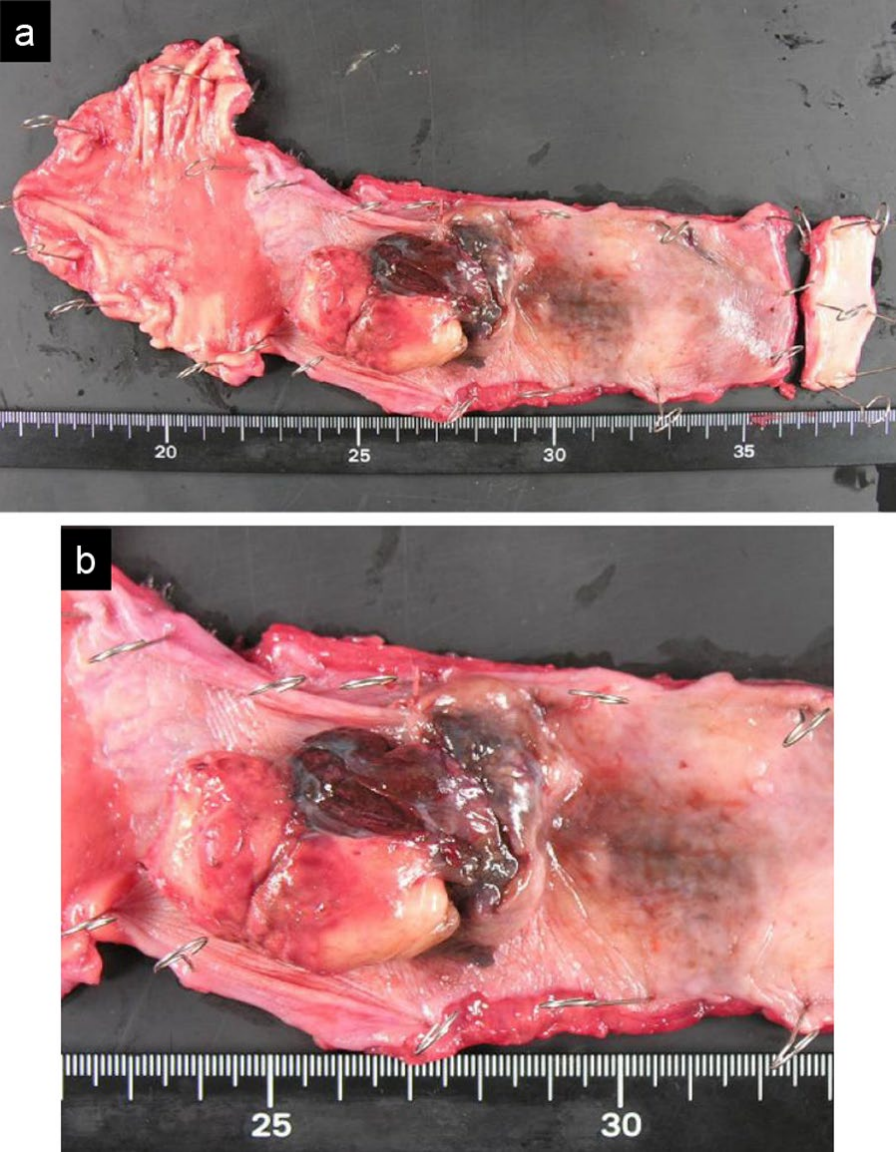
**a** Resected specimen after thoracoscopic subtotal esophagectomy. **b** Magnified view of the same specimen described in **a**. An elevated black mass is found in the esophagus

**Fig. 2:**
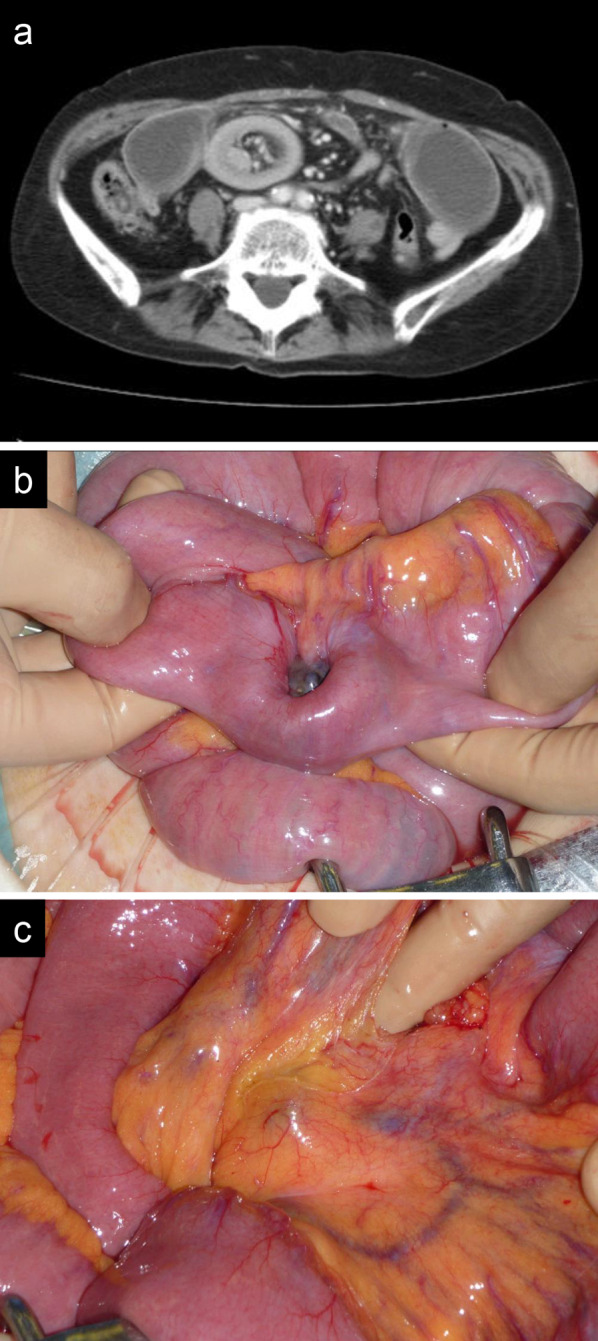
**a** Computed tomography showing the presence of an intestinal invagination. **b** Intraoperative findings. After repositioning the invagination, a black tumor is found in the jejunum. **c** Intraoperative findings. A black lymph node is found in the region of the second jejunal artery

**Fig. 3:**
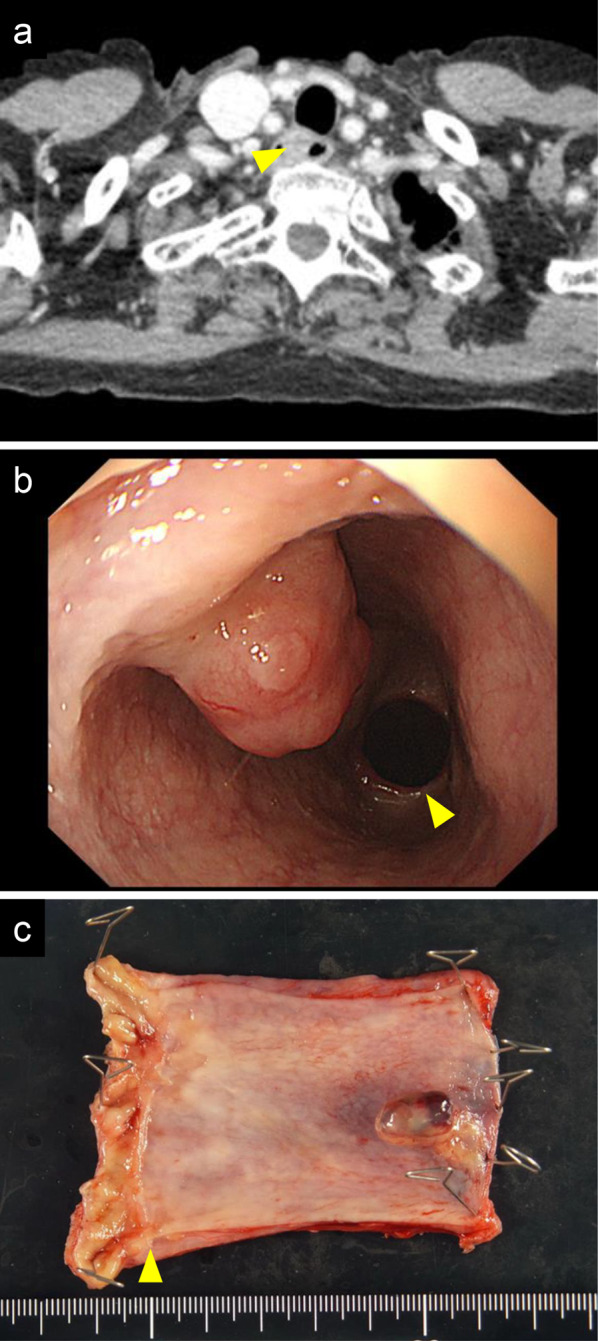
**a** Computed tomography showing a mass in the cervical esophagus. The yellow arrowhead indicates a mass found in the cervical esophagus. **b** Endoscopy showing the elevated lesion in the cervical esophagus. The yellow arrowhead indicates the site of esophagogastric anastomosis. **c** Resected specimen after cervical esophagectomy. A black mass is found in the cervical esophagus. The yellow arrowhead indicates the esophagogastric anastomosis

A 65-year-old female patient with PMME underwent thoracoscopic subtotal esophagectomy, gastric tube reconstruction via the posterior mediastinal route, and cervical esophagogastric anastomosis. The postoperative course was uneventful. Histopathological examination of the resected specimen (Fig. 1) revealed that the tumor was PMME with tumor invasion into the muscularis propria, and no lymph node metastasis was found in 57 harvested lymph nodes. At the age of 68 years, she developed an acute small obstruction due to invagination (Fig. 2a) and underwent laparotomy. An intestinal invagination was found in the jejunum 70 cm from the ligament of Treitz, which was repositioned using Hutchinson’s maneuver, and a black tumor (Fig. 2b) was found at the leading point. Another black tumor was found 20 cm proximal, and a black lymph node (Fig. 2c) was found along the second jejunal artery. The jejunum and mesentery were resected, and the black lymph nodes were dissected. Histopathological examination of the resected specimen revealed two metastatic malignant melanomas in the jejunum and one metastatic node in the five dissected mesenteric nodes. Although there was no sign of recurrence on semiannual computed tomography (CT) follow-up for 7 years, at the age of 75 years, CT showed a mass in the cervical esophagus (Fig. 3a). Endoscopy revealed an elevated lesion in the cervical esophagus (Fig. 3b), and biopsy revealed a recurrent malignant melanoma. After obtaining informed consent, we scheduled the surgery. She underwent thoracoscopic mobilization of the gastric tube and esophagus followed by cervical esophagectomy (Fig. 3c). Reconstruction was performed by cervical esophageal jejunal anastomosis and jejunal gastric anastomosis using a free jejunum flap in the third jejunal arteriovenous region. The jejunal artery and jejunal vein were anastomosed to the transverse cervical artery and internal jugular vein, respectively. The postoperative course was uneventful. No BRAF mutations (V600E and V600K) were detected in the tumor. The programmed death-ligand 1 (PD-L1) expression and microsatellite instability (MSI) status in the tumor were < 1% and microsatellite stable, respectively.

The patient received 24 courses of nivolumab therapy for one year as a postoperative adjuvant therapy. Subsequently, at the age of 78 years (18 months after the completion of adjuvant nivolumab therapy), CT showed an enlarged left cervical lymph node and a mass in the right lower lobe of the lung. She underwent left cervical lymph node dissection and thoracoscopic wedge resection of the right lower lobe of the lung. Histopathological examination revealed that both tumors were metastases from malignant melanoma. At the age of 79 years (14 years since the initial diagnosis), she received eight courses of nivolumab therapy as a second postoperative adjuvant therapy with no sign of recurrence in a 9-month follow-up period after the third metastasectomy.

## Discussion

We report a case of PMME with metastatic recurrence treated with three metastasectomies and subsequent nivolumab therapy. Although there have been few reports on PMME owing to its rarity, summary reports of more than 50 cases have shown a poor prognosis with a median survival time of 13.5–16 months [2, 3]. Dai et al. reported a median disease-free survival of 5.9 months for 70 resected cases [3], and Wang et al. reported a median relapse-free survival (RFS) of 4.5 months for 58 resected cases [1], indicating that the prognosis for resected cases was also poor. Although many papers, including our previous report [10], have reported on resection as the initial treatment of PMME, there are few reports describing treatment after recurrence [6–8, 11–14]. According to previous reports, recurrence sites vary widely, and treatments such as surgery, chemotherapy, radiation therapy, and immunotherapy are used. In recent reports, nivolumab and ipilimumab have been administered as immunotherapy [6–8]. Thus, the treatment for recurrence after primary resection of PMME has not yet been determined. The NCCN guidelines for cutaneous melanoma state that resection should be considered in cases of resectable metastatic recurrent disease [5]. In cases of unresectable metastatic recurrence, systemic therapy with immune checkpoint inhibitors or BRAF inhibitors in the presence of BRAF mutations is recommended [5]. In contrast, the UK national guidelines for head and neck mucosal melanoma recommend systemic therapy for locally recurrent lesions, and the decision to resect should be made on a case-by-case basis [9]. However, gastrointestinal (GI) melanoma has a poorer prognosis than cutaneous melanoma [15], and the prognosis of PMME is even poorer [16]. Zheng et al. analyzed the prognostic factors in 1080 cases of GI melanoma. In a multivariate analysis, they reported that resection of the primary site is an independent prognostic factor for overall survival and cancer-specific survival, and that decreased survival was associated with esophageal origin and no resection of the primary site [16]. Unfortunately, the efficacy of nivolumab in advanced mucosal melanoma is not comparable to that in cutaneous melanoma [17, 18]. Taken together, despite the advances in immune checkpoint inhibitors, surgical resection plays an essential role in the treatment of PMME. Therefore, aggressive surgery should be considered for single or oligometastasis of PMME where surgical expertise is available.

The postoperative follow-up of PMME patients has not been determined. Dai et al. reported that more than 90% of recurrences occur within 2 years after surgery for PMME. The recurrence sites were lymph nodes, liver, lung, bone, and anastomosis in order of frequency [3]. They followed up with CT every 3 months until the second postoperative year and every 6 months from the third to the fifth postoperative year. In this case, we followed up with CT every 3 months until the second postoperative year, and every 6 months after the third postoperative year. The endoscopy was performed once a year. After the fifth postoperative year, we have continued to follow-up with CT every 6 months.

The NCCN guidelines for cutaneous melanoma recommend resection combined with adjuvant therapy, including nivolumab, for both resectable primary and recurrent lesions. Wang et al. reported significantly prolonged RFS in PMME patients treated with surgery combined with adjuvant therapy, such as temozolomide/dacarbazine-based chemotherapy or high-dose interferon therapy, versus surgery alone [1]. They also reported the use of anti-PD-1 antibodies in 12 patients with PMME and a significantly prolonged progression-free survival (PFS) in the anti-PD-1 antibody group compared to the chemotherapy group using temozolomide/dacarbazine or paclitaxel together with carboplatin [1]. In addition to malignant melanoma, the efficacy of nivolumab has been reported in unresectable advanced or recurrent non-small cell lung cancer [19, 20], head and neck squamous cell carcinoma [21], esophageal squamous cell carcinoma [22, 23], gastric cancer [24], microsatellite instability-high colorectal cancer [25], malignant pleural mesothelioma [26], and classical Hodgkin's lymphoma [27]. Its efficacy has also been reported in postoperative adjuvant therapy for esophageal cancer [28] and urothelial carcinoma [29]. Even in cases of PMME, it is certainly better to use adjuvant nivolumab therapy after the resection of resectable primary lesions or metastases/recurrences regardless of the PD-L1 expression and the status of MSI [30, 31].

Nomura et al. reported that the efficacy of the second nivolumab therapy in patients with melanoma was associated with the duration of PFS after the first nivolumab therapy [32]. Our patient, who did not experience recurrence for 18 months after completion of the first adjuvant nivolumab therapy, is considered a good candidate for second adjuvant nivolumab therapy, although evidence for this is still lacking.

## Conclusions

Although the prognosis of patients with metastatic recurrence of PMME is poor, aggressive surgical intervention followed by adjuvant therapy with nivolumab may lead to long-term survival in the case of single or resectable oligometastatic lesions.

## Data Availability

Not applicable.

## Abbreviations

PMME: Primary malignant melanoma of the esophagus
PD-1: Programmed death 1
NCCN: National Comprehensive Cancer Network
UK: United Kingdom
CT: Computed tomography
GI: Gastrointestinal
RFS: Relapse-free survival
PFS: Progression-free survival
MSI: Microsatellite instability
PD-L1: Programmed death-ligand 1
